# Melatonin Counteracts Drought Induced Oxidative Damage and Stimulates Growth, Productivity and Fruit Quality Properties of Tomato Plants

**DOI:** 10.3390/plants9101276

**Published:** 2020-09-28

**Authors:** Mohamed F. M. Ibrahim, Ola H. Abd Elbar, Reham Farag, Mohamed Hikal, Amr El-Kelish, Ahmed Abou El-Yazied, Jawaher Alkahtani, Hany G. Abd El-Gawad

**Affiliations:** 1 Department of Agricultural Botany, Faculty of Agriculture, Ain Shams University, 11566 Cairo, Egypt; olaabdelbar@agr.asu.edu.eg (O.H.A.E.); Reham_hassan@agr.asu.edu.eg (R.F.); 2 Department of Biochemistry, Faculty of Agriculture, Ain Shams University, 11566 Cairo, Egypt; Mohamed_elsayed4@agr.asu.edu.eg; 3 Botany Department, Faculty of Science, Suez Canal University Ismailia, 41522 Ismailia, Egypt; 4 Department of Horticulture, Faculty of Agriculture, Ain Shams University, 11566 Cairo, Egypt; ahmed_abdelhafez2@agr.asu.edu.eg; 5 Department of Botany and Microbiology, College of Science, King Saud University, Riyadh 11451, Saudi Arabia; jsalqahtani@ksu.edu.sa

**Keywords:** Melatonin, *Solanum lycopersicum* L., drought, antioxidant enzymes, quality attributes

## Abstract

Melatonin “*N*-Acetyl-5-methoxytryptamine” (MT) has recently been considered as a new plant growth regulator with multiple physiological functions. Although many previous studies have confirmed that exogenous applied-MT can alleviate the deleterious effects of drought stress in many plant species, most of these studies were exclusive on seeds, seedlings, and young plants for a short period of their life cycles. Therefore, the knowledge of using MT as a potential promising agricultural foliar application to improve crop productivity and quality is still insufficient under adverse open field conditions. In this study, we investigated the effect of MT as a foliar application at 0, 20, and 40 ppm on tomato plants that were grown in the open field under the long term of optimal and deficit irrigation conditions. The results indicated that exogenous MT significantly enhanced plant growth, chlorophyll and activities of antioxidant enzymes, including ascorbate peroxidase (APX), catalase (CAT), and peroxidase (POX). This improvement was associated with a marked reduction in proline and soluble sugars. In addition, applied-MT worked as a protective agent against oxidative damage by reducing the cellular content of toxic substances such as H_2_O_2_ and malondialdehyde (MDA). Similarly, MT-treated plants showed greater total fruit yield with improving its quality attributes like total soluble solids (TSS), ascorbic acid, and lycopene. Generally, the highest significant fruit yield either under well-watered (13.7%) or water deficit (37.4%) conditions was achieved by the treatment of 20 ppm MT. These results indicate that exogenous MT played an essential role in enhancing tomato tolerance to deficit irrigation and could be recommended as a promising agricultural treatment under such conditions.

## 1. Introduction

Water deficit is thought to be one of the environmental factors that dramatically restrict plant growth, development, and yield [1,2,3,4]. It causes several interconnected responses leading to considerable damages at morphological, physiological, biochemical, and molecular levels [5,6,7,8,9]. Among these effects, water deficit causes altering of leaf water status, hindering stomatal conductance, reducing nutrient uptake, degradation of leaf pigments and photosynthesis depression [9,10,11]. Furthermore, the rapid accumulation of reactive oxygen species (ROS) and drought induced oxidative damages have been extensively reported in many previous studies [12,13,14]. However, plants as sessile organisms possess a broad spectrum of defense strategies to cope with such conditions. These responses begin with a cascade of complex signal perception and transduction systems that comprise specific osmosensors such as phospholipases, kinases and secondary messengers [15,16]. These steps are essential to modulate a wide array of plant responses. These influences include the development of enzymatic and non-enzymatic antioxidative systems, alteration of endogenous phytohormones, accumulation of osmolytes and expression of specific stress-related genes [17,18,19,20].

Tomato (*Solanum lycopersicum* L.) is the second most widely cultivated vegetable crop after potatoes that is extensively consumed worldwide [21]. It is considered a natural medicine with multiple health benefits for humans because it contains a significant proportion of vitamins, essential amino acids, sugars, minerals, dietary fibers, and antioxidants that prevent—or reduce the opportunities of—different cancer diseases [22]. Most of the commercial tomato cultivars are sensitive to water deficit, specifically at seed germination and early seedling growth stages [23]. On the contrary, exposing tomato plants to a short period of water deficit, specifically during the flowering stage, may lead to significant improvement in the yield and quality of the produced fruits compared to the stressed plants in the other developmental stages [24]. Generally, the negative effect of drought stress depends on the growth stage of the plant and exposure period to water deficit.

Melatonin is a new multifunctional plant growth regulator [25]. It can regulate a wide array of plant processes at developmental, physiological, biochemical, and molecular levels [20,26,27,28,29]. It has the potential to enhance plant morphogenesis in vitro [30], seed germination, plant growth, and rhizogenesis, and delay leaf senescence in vivo [28,29,31,32]. Furthermore, MT plays a pivotal role in plant protection against biotic [33] and diverse environmental stresses, i.e., drought [11,20,28], salinity [27,34], chilling [35], heat stress [36], and heavy metals [37,38]. Many possibilities could be suggested to explain how MT can help plants to alleviate the adverse effects of various environmental conditions. One of the most important defensive mechanisms in this respect is the protection of the photosynthetic apparatus via improving the scavenging efficiency of reactive oxygen species (ROS) and reducing the stress induced oxidative damages [10,20,39]. Under drought stress, MT join in the readjustment of the cell osmotic potential and accumulation of osmolytes such as proline and soluble sugars [10,40]. Moreover, MT can maintain the water status of water-stressed plants through regulating stomatal movement [41] and modulating a broad spectrum of anatomical aspects i.e., preserving the integrity of cell membranes [42] and increasing the cuticle and/or wax accumulation [43,44]. In addition, it has been confirmed that during the exposure to stress, MT has a close linkage in plant signal transduction and can trigger cascades of reprogramming primary metabolites, transcriptomes, and proteomes [45].

Melatonin was recommended to mitigate drought stress in several plant species, including apple [46], tomato [47], maize [10], and coffee [39]. However, most of these previous studies were limited to young plants or seedlings that were exposed to short periods of water stress. Such studies are not enough to be taken as a valid recommendation or as an agricultural application to improve crop production under the long-term conditions of water shortage and open field conditions. Therefore, this study was conducted to fill this gap of knowledge by comparing the effect of foliar applications of MT on growth, chlorophyll, accumulation of osmolytes, oxidative damage, antioxidant enzymes, fruit yield and its quality attributes including total soluble solids (TSS), ascorbic acid and lycopene of tomato plants under both long-term optimum water and water deficit conditions.

## 2. Material and Methods

### 2.1. Plant Material and Growth Conditions

The field experiment was carried out during the two growing seasons of 2018 and 2019 at the Experimental Farm, Faculty of Agriculture, Ain Shams University, Shoubra El-Kheima, Egypt, to investigate the effect of foliar application of melatonin (MT) at 0 (distilled water), 20 and 40 ppm on tomato plants grown under two different water regimes: well-watered (after the depletion of 55–60% of available soil water) and water deficit (after the depletion of 85–90% of available soil water). Seeds of the tomato plant (023 F_1_) produced by Sakata Vegetables, Europe, France, were sown under greenhouse conditions (25 °C ± 5), 16 h light and 8 h dark cycles, in trays with 50 individual cells (4 × 4 × 6) containing sandy-loam soil (2:1 *w*/*w*). Five-week-old tomato transplants were set up into the experimental soil (clay loam) on the 30th of April 2018 and 2019 seasons. The chemical properties of the experimental soil are presented in Table 1.

The area of the experimental plot was 17.5 m^2^ consisted of five ridges; each ridge was 3.5 m length and 1 m width. The plant distance was 40 cm apart on one ridge, an alley (2 m wide) was left as a border between both irrigation regimes.

Calcium super-phosphate (15% P_2_O_5_) at 720 kg ha^−1^ was banded on ridges at two times, the first (480 kg) was added during the soil preparation, and the second one (240 kg) was carried out at the flowering stage. Ammonium nitrate (33% N) at 600 kg.ha^−1^ and potassium sulfate (48% K_2_O) at 360 kg ha^−1^ were applied in two portions; the first was applied after two weeks from transplanting, and the second one was carried out after one month from the first addition. Agricultural management, disease, and pest control programs were followed according to the recommendations of the Egyptian Ministry of Agriculture.

### 2.2. Treatments and Experimental Design

Two Irrigation regimes were started two weeks after transplanting (0 time): the first one was resumed after the depletion of 55–60% of available soil water (well-watered) and the second was resumed after the depletion of 85–90% of available soil water (water-deficit). Soil samples from the experimental site were taken before soil preparation by an Auger T-Handle at depths of 0–20 and 20–40 cm from the soil surface to determine the field capacity, wilting point, and available soil water during the two seasons (Table 2).

After that, the soil moisture content was regularly estimated by the weight method to determine the timing of irrigation treatments. The samples were dried at 105 °C for 24 h and re-weighting until it reached a constant weight. The percentage of soil moisture was measured using the following equation:

$$\mathrm{Soil}\;\mathrm{moisture}\%\;=\;\frac{\mathrm{Weight}\;\mathrm{before}\;\mathrm{drying}\;-\mathrm{Weight}\;\mathrm{after}\;\mathrm{drying}}{\mathrm{Weight}\;\mathrm{after}\;\mathrm{drying}}\;\times 100$$

The leaves of tomato plants were sprayed with 0 (distilled water as a check), 20 and 40 ppm MT (Bio Basic, Markham, ON, Canada) two times: after 30 and 50 days from transplanting (vegetative stage). Tween 20 at 0.05 mL L^−1^ was used as a wetting agent for all foliar treatments. The experimental design was split-plot with three replicates. Irrigation regimes were assigned in the main plots, and the foliar applications of MT were distributed in the sub-plots.

### 2.3. Data Recorded

#### 2.3.1. Vegetative Growth

Shoot fresh and dry weights were determined after sampling at 65 days from transplanting (flowering stage). To determine the dry weight, the samples were cleaned by washing with tap water, then dried in an air-forced ventilated oven at 70 °C until constant weight.

#### 2.3.2. Chlorophyll, Proline and Soluble Sugars

After 65 days from transplanting, leaves (the 4th and 5th from the top) of tomato plants were collected randomly for the biochemical analyses. Total chlorophyll was extracted in 80% acetone and estimated according to the method of Arnon [48] using the following equation:

Total chlorophyll (µg/mL) = (20.2 × O.D at 645 nm) + (8.02 × O.D at 663 nm).

where O.D is the optical density.

Proline concentration was determined using ninhydrin reagent, as described by Bates et al. [49]. Total soluble sugars were estimated by the phenol sulphuric acid method, as described by Chow and Landhäusser [50].

#### 2.3.3. Hydrogen Peroxide, DAB Staining, and Lipid Peroxidation

Hydrogen peroxide (H_2_O_2_) concentration was determined, according to Velikova et al. [51] with some modifications. Leaf samples were homogenized in tri-chloroacetic acid (TCA). The homogenate was centrifuged at 10,000 rpm and 4 °C for 10 min. Subsequently, 0.75 mL of the supernatant was added to 0.75 mL of 10 mM K-phosphate buffer (pH 7.0) and 1.5 mL of 1 M KI. H_2_O_2_ concentration was evaluated by comparing its absorbance at 390 nm to a standard calibration curve.

DAB staining was carried out as described by Wei, Li, Chu, Reiter, Yu, Zhu, Zhang, Ma, Lin and Zhang [29]; the terminal leaflet of the 4th compound leaf from the top from each treatment was ditched and soaked immediately for 24 h in a solution contains 50 mM Tris-HCl buffer, pH 4.0, and diaminobenzidine (DAB) at 1 mg/mL solution. After 24 h, leaves were transferred to absolute alcohol to remove the leaf pigments. The last step was repeated several times with absolute alcohol until removing all leaf pigments. The brown color indicates the existence of H_2_O_2_.

Lipid peroxidation was measured by the determination of malondialdehyde (MDA), as described by Heath and Packer [52]. Leaf tissues (the 4th leaf from the top) were homogenized in 0.1% (*w*/*v*) trichloroacetic acid (TCA). The homogenate was centrifuged at 4500 rpm for 15 min. The reaction mixture contained 1 mL from the supernatant and 4 mL 0.5% (*w*/*v*) thiobarbituric acid (TBA) dissolved in 20% (*w*/*v*) TCA. The mixture was heated in boiling water for 30 min then the mixture was cooled at room temperature and centrifuged at 4500 rpm for 15 min. The absorbance (A) of the supernatant was measured at 535 and corrected for non-specific turbidity at 600 nm using a spectrophotometer (Chrom Tech CT-2200; Taiwan). The MDA concentration (nmol g^−1^ FW) was calculated using Δ OD (A532-A600) and the extinction coefficient (ε = 155 mM^−1^ cm^−1^).

#### 2.3.4. Enzyme Assays

After 65 days from transplanting, leaf tissue of tomato plants (0.5 g) was taken from the 4th leaf and homogenized in 4 mL 0.1 M K-phosphate buffer (pH 7.0) containing 1% (*w*/*v*) polyvinylpyrrolidone (PVP) and 0.1 mM EDTA. The homogenate was centrifuged at 10,000 rpm for 15 min, and the supernatant was used as a crude enzyme extract. All steps in the preparation of the enzyme extract were carried out at 0–4 °C. Total soluble protein was determined, according to Bradford [53].

##### Ascorbate Peroxidase (APX) Assay

Ascorbate peroxidase (APX) (EC 1.11.1.11) activity was measured according to the method of Nakano and Asada [54], by monitoring the decrease in absorbance at 290 nm following the ascorbate oxidation for 3 min using a spectrophotometer (Chrom Tech CT-2200; Taiwan). The reaction mixture with a total volume of 3 mL included 100 μL crude enzyme, 50 mM phosphate buffer (pH 7), 0.1 mM EDTA, 0.5 mM ascorbic acid, and 0.1 mM H_2_O_2_. The reaction was initiated by the addition of H_2_O_2_. One unit of enzyme activity of APX was defined as the amount of enzyme required for oxidation of 1 μmol of ascorbate per minute. The rate of ascorbate oxidation was calculated using the extinction coefficient (ε = 2.8 mm^−1^ cm^−1^). The enzyme activity was expressed as unit.mg^−1^ protein.

##### Catalase (CAT) Assay

Catalase (CAT) (EC 1.11.1.6) activity was determined according to the method of Cakmak et al. [55]. The CAT activity was measured by monitoring the decrease in absorbance at 240 nm following the decomposition of H_2_O_2_ for 1 min using a spectrophotometer (Chrom Tech CT-2200; Taiwan). The reaction mixture with a total volume of 3 mL contained 15 mM H_2_O_2_ in 50 mM phosphate buffer (pH = 7). The reaction was initiated by adding 50 μL crude enzyme. The activity was calculated from the extinction coefficient (ε = 40 mM^−1^ cm^−1^) for H_2_O_2_. One unit of enzyme activity was defined as the decomposition of 1 μmol of H_2_O_2_ per minute. The CAT activity was expressed as unit.mg^−1^ protein.

##### Peroxidase (POX) Assay

Peroxidase (EC1.11.1.7) activity was quantified by the method of Hammerschmidt et al. [56]. The assay mixture (100 mL) contained 10 mL of 1% (*v*/*v*) guaiacol, 10 mL of 0.3% H_2_O_2_ and 80 mL of 50 mM phosphate buffer (pH = 6.6). The volume of 100 μL of the crude enzyme was added to 2.9 mL of the assay mixture to start the reaction. The absorbance was recorded every 30 s for 3 min at 470 nm using a spectrophotometer (Chrom Tech CT-2200; Taiwan). The rate of change in absorbance per minute was calculated, and one unit of the enzyme was expressed as ΔOD = 0.01. The APX activity was expressed as unit.mg^−1^ protein.

#### 2.3.5. Fruit Yield, TSS, Ascorbic Acid and Lycopene

Five plants were randomly selected and labeled from each plot (from the inner ridges), tomato fruits were harvested several times from the labeled plants. The average of fruit yield/ha was calculated using the following equation:

$$\mathrm{The}\;\mathrm{average}\;\mathrm{of}\;\mathrm{fruit}\;\mathrm{yield}\;(\mathrm{ton}/\mathrm{ha})\;=\;\frac{\mathrm{The}\;\mathrm{average}\;\mathrm{weight}\;\mathrm{of}\;\mathrm{fruit}\;\mathrm{yield}\;\left(g/\mathrm{plant}\right)\times 25,000}{1,000,000}$$

where 25,000 is the number of plants/ha., and 1,000,000 is the metric conversion calculator from g to ton.

The total soluble solids (TSS) were determined using a hand refractometer (OPTIKA, HR-190). Ascorbic acid was determined using the 2,6-Dichloroindophenol titrimetric method according to A.O.A.C [57]. Lycopene content was measured as described by Fish et al. [58] with some modifications. Five grams were taken from six fruits without seeds and homogenized in 50 mL of a mixture containing hexane–acetone–ethanol (2:1:1, *v*/*v*) for 1 min. After that, 15 mL of water was added and the samples were vortexed for 15 s. Following phase separation on ice, lycopene concentration was determined by measuring the absorbance of the organic phase (hexane) at 503 nm (Chrom Tech CT-2200; Taiwan). All the procedures were performed under dim lighting. Lycopene content was calculated using the molar extinction coefficient of 17.2 mol^−1^ cm^−1^.

### 2.4. Statistics

A two-way ANOVA procedure was followed using SAS [59] software. Means were calculated from three replicates, and the Duncan multiple range test (*p* ≤ 0.05) was used to determine the significant differences between means.

## 3. Results

### 3.1. Changes in the Vegetative Growth and Total Chlorophyll Concentration

**Figure 1:**
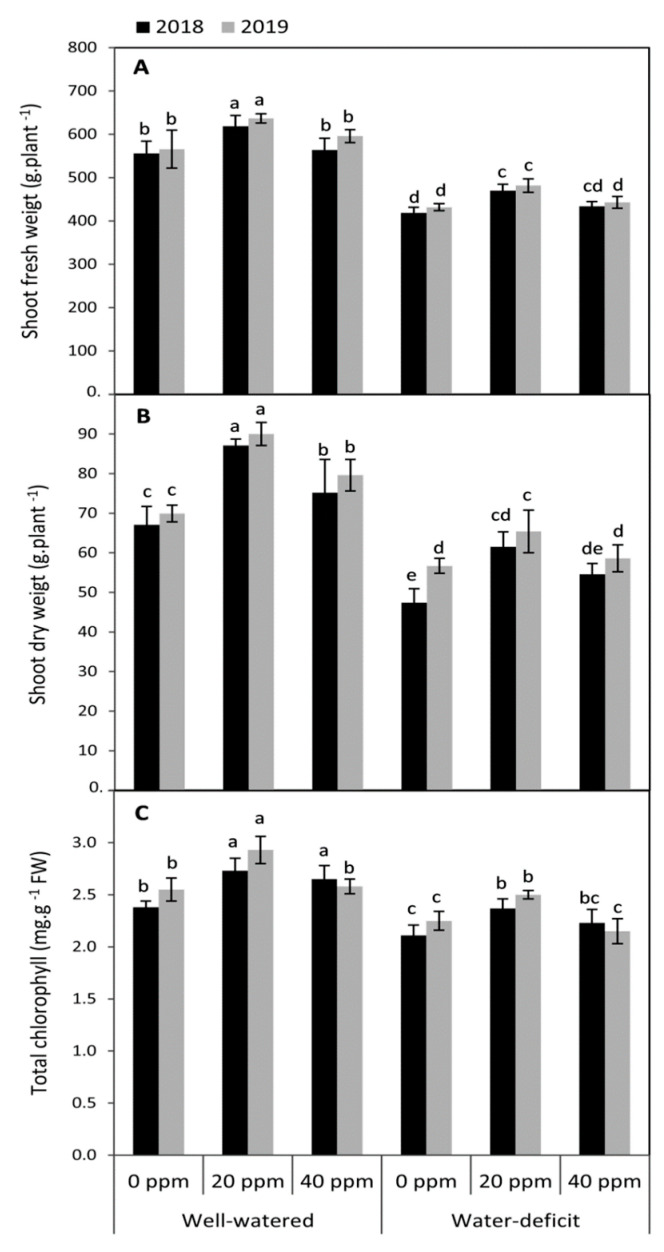
Effect of foliar application of melatonin (MT) at 0, 20 and 40 ppm on shoot fresh weight (**A**), shoot dry weight (**B**), and total chlorophyll (**C**) of tomato plants grown under well-watered and water deficit conditions in the seasons of 2018 and 2019. Means of three replicates were presented ±SD. Different letters are significant differences, according to Duncan’s multiple range tests (*p* < 0.05).

Plants that were exposed to deficit irrigation demonstrated a significant (*p* ≤ 0.05) decrease in the vegetative growth in terms of shoot fresh weight and shoot dry weight compared to the well-irrigated plants in both seasons (Figure 1). As well as, the water-stressed plants had a significant lower chlorophyll concentration than the well-irrigated plants in both seasons. When MT was applied especially at 20 ppm as a foliar application, shoot fresh weight, shoot dry weight and total chlorophyll were significantly improved compared to MT-untreated plants either under full or deficit irrigation conditions in both seasons. However, except the shoot dry weight under well watered conditions in both seasons and total chlorophyll under well watered conditions in the first season, there were no significant differences between the MT-untreated plants and those that were treated by 40 ppm MT under the two levels of irrigations in both seasons. Overall, under well-irrigated conditions, the highest significant increases in shoot fresh weight (112.5%), shoot dry weight (129.3%) and total chlorophyll (114.8%) were achieved by the treatment of 20 ppm MT when compared to the MT-untreated plants whereas; these values reached about 111.9, 122.5% and 111.7% for the same traits, respectively, under water deficit conditions (averages of two seasons).

### 3.2. Changes in the Compatible Solutes (Proline and Soluble Sugars)

**Figure 2:**
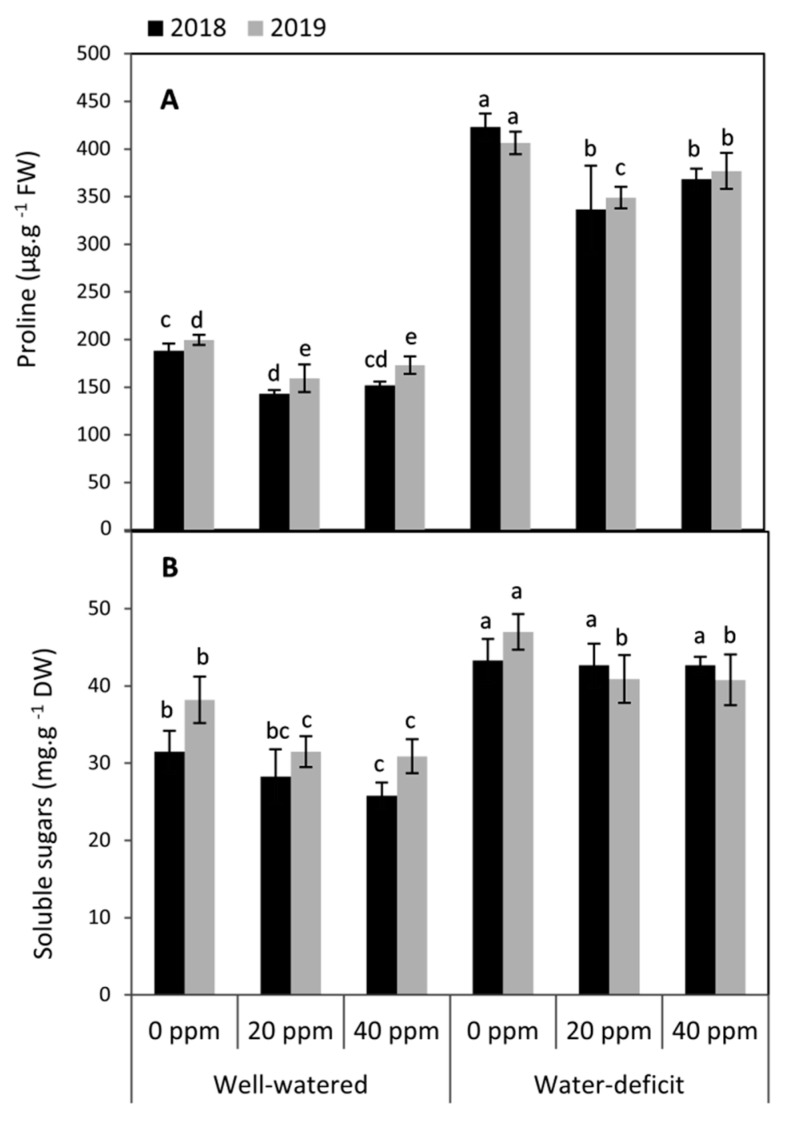
Effect of foliar application of melatonin (MT) at 0, 20 and 40 ppm on proline (**A**) and soluble sugars (**B**) of tomato plants grown under well-watered and water deficit conditions in the seasons of 2018 and 2019. Means of three replicates were presented ± SD. Different letters are significant differences, according to Duncan’s multiple range tests (*p* < 0.05).

Water restriction significantly (*p* ≤ 0.05) elevated proline and soluble sugars compared to the well-irrigated conditions in both seasons (Figure 2). Conversely, MT applied at 20 or 40 ppm obviously diminished these molecules under well-watered conditions. This trend was also conspicuous under water stress conditions for proline in both seasons and soluble sugars in the second season, respectively. Generally, plants that were treated by 20 ppm had lower proline by 22.1 and 17.3% compared to the MT-untreated plants under well-watered and deficit conditions, respectively, whereas the lowest concentration of soluble sugars (17.8 and 7.2%) was achieved by the treatment of MT at 40 ppm when compared to the MT-untreated plants under well and deficit irrigation, respectively (averages of the two seasons).

### 3.3. Evaluation of the Oxidative Damage in Leaf Tissues

**Figure 3:**
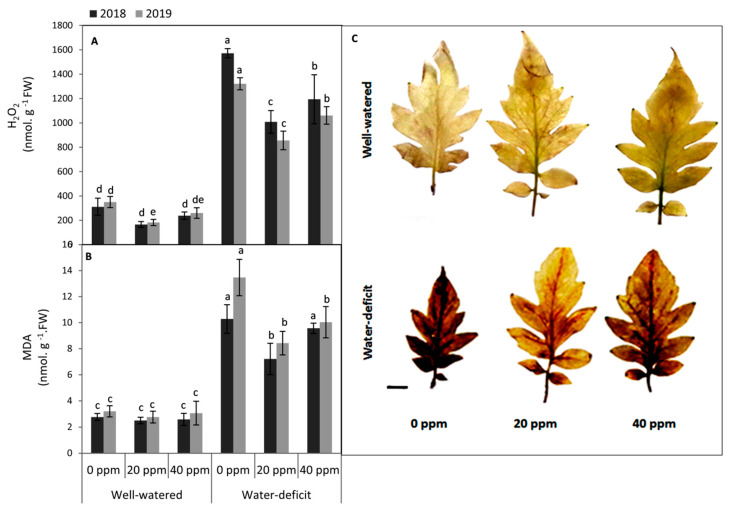
Effect of foliar application of melatonin (MT) at 0, 20 and 40 ppm on (**A**) the concentration of H_2_O_2_, (**B**) lipid peroxidation as indicated by malondialdehyde (MDA) and (**C**) H_2_O_2_ distribution using DAB staining in the terminal leaflet of the 4th leaf of tomato plants grown under two different irrigation regimes (Brown DAB staining color indicates accumulation of H_2_O_2_. Bar = 1 cm). Means of three replicates were presented ±SD. Different letters are significant differences, according to Duncan’s multiple range tests (*p* < 0.05).

The oxidative damage in leaf tissues of the water deficit stressed plants in terms of the significant increase in the concentration of H_2_O_2_, elevating the rate of lipid peroxidation as indicated by MDA and showing a deeper brown color with DAB staining (Figure 3) were observed in both seasons. Under well-watered conditions, H_2_O_2_ in the first season and MDA in both seasons did not show any significant differences between MT-treated (20 and 40 ppm) and non-treated plants. The effect of foliar applied-MT on the concentration of H_2_O_2_ and MDA was more obvious under water deficit conditions in both seasons. Relative to the non-treated plants, the treatment of 20 ppm MT reduced H_2_O_2_ and MDA to 64.5 and 66.4%, respectively, under water stress conditions (average of two seasons). Furthermore, MT—applied either at 20 or 40 ppm—exhibited an obvious decrease in the browning of leaf tissues by DAB staining compared to the untreated plants, which means minimizing the concentration of H_2_O_2_ in leaf tissues. Generally, MT applied at 20 ppm was more effective than 40 ppm in this respect.

### 3.4. Changes in the Activities of Antioxidant Enzymes

**Figure 4:**
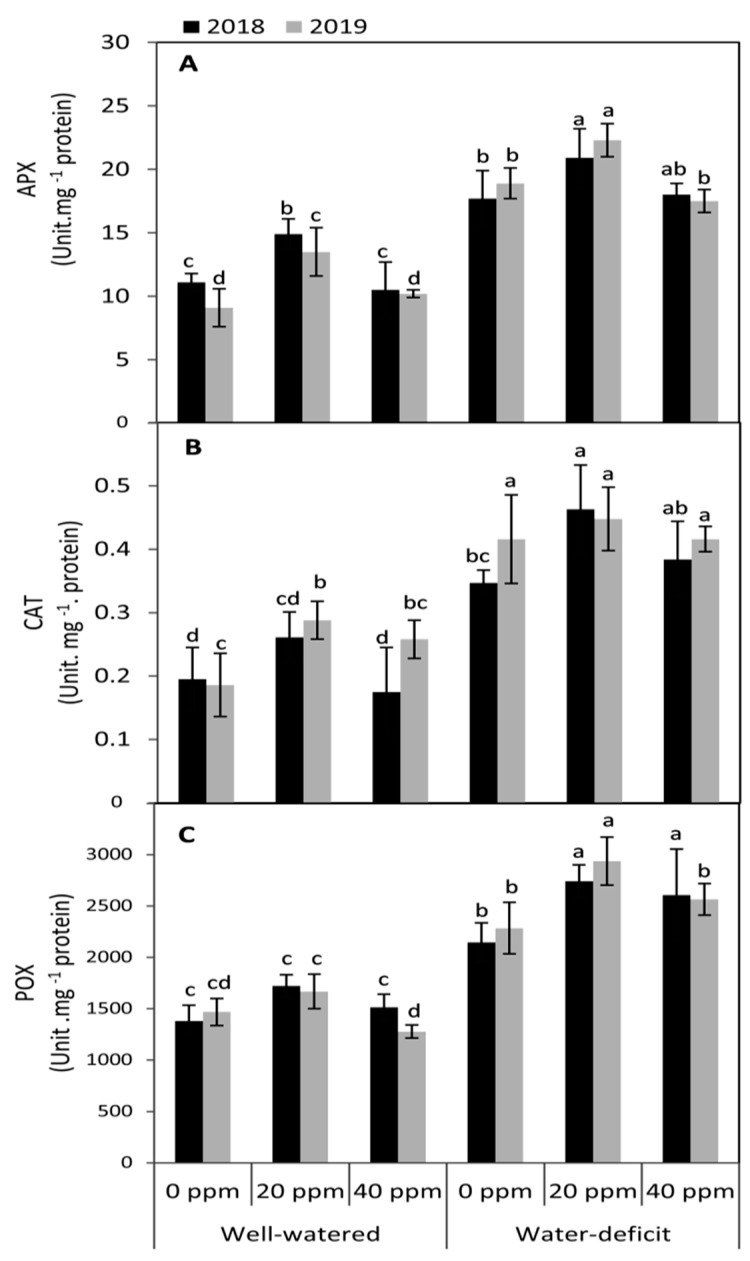
Effect of foliar application of melatonin (MT) at 0, 20 and 40 ppm on the activities of antioxidant enzymes; ascorbate peroxidase (POX; **A**), catalase (CAT; **B**) and peroxidase (POX, **C**) of tomato plants grown under well-watered and water deficit conditions in the seasons of 2018 and 2019. Means of three replicates were presented ±SD. Different letters are significant differences, according to Duncan’s multiple range tests (*p* < 0.05).

Under water stress conditions, all studied antioxidant enzymes, including ascorbate peroxidase (APX), catalase (CAT) and peroxidase (POX), demonstrated higher activities when compared to the well-irrigated plants in both seasons (Figure 4). The highest significant values for all studied antioxidant enzymes were achieved by the treatment of 20 ppm MT compared to the untreated plants in both seasons. These increases amounted by 118, 120.6 and 128.1% for APX, CAT and POX, respectively, over the MT-untreated plants (averages of two seasons). However, under optimum irrigation conditions, plants treated with MT at 20 ppm showed relatively higher significant (*p* ≤ 0.05) activities in APX in both seasons than the MT-untreated plants. This trend was obvious in respect to CAT in the second season; while no significant changes were observed in the activity of POX between all MT-treated and untreated plants in both seasons.

### 3.5. Changes in Fruit Yield and Quality Attributes

**Figure 5:**
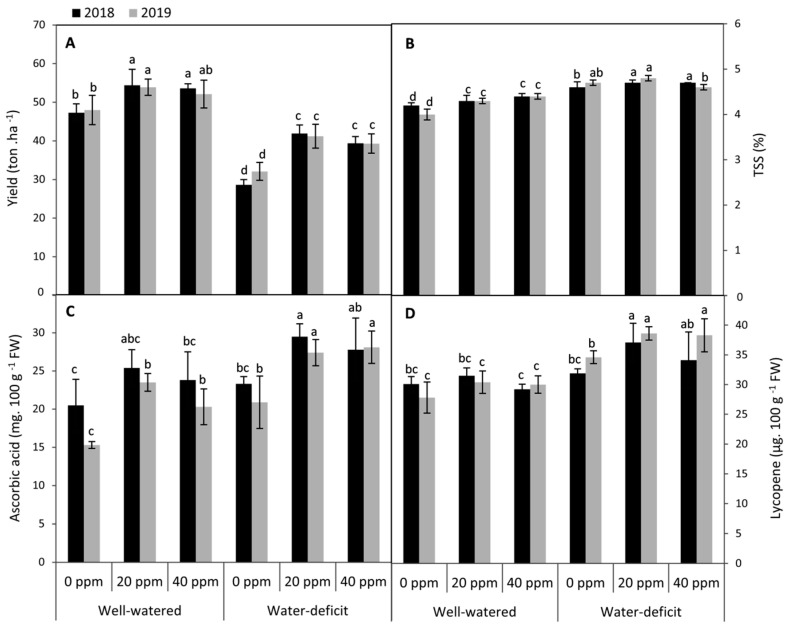
Effect of foliar application of d melatonin (MT) at 0, 20 and 40 ppm on (**A**) fruit yield, (**B**) total soluble solids (TSS), (**C**) ascorbic acid and (**D**) lycopene of tomato plants grown under well-watered and water deficit conditions in the seasons of 2018 and 2019. Means of three replicates were presented ±SD. Different letters are significant differences, according to Duncan’s multiple range tests (*p* < 0.05).

Data in Figure 5 show that the fruit yield of tomato plants showed a significant (*p* ≤ 0.05) decrease by exposure to water deficit treatment compared to the well-watered plants in both seasons. Melatonin applied at 20 or 40 ppm significantly improved the eventual fruit yield of tomato plants compared to the untreated plants under both investigated levels of irrigation in both seasons. In this respect, the highest significant quantities of fruit yield either under well-watered (13.7%) or water deficit (37.4%) conditions were achieved by the treatment of 20 ppm MT (averages of two seasons). These results indicate that applied-MT was more effective under water restriction conditions than the optimum ones. Furthermore, relative to the lower concentration of MT at 20 ppm, the other treatment of MT at 40 ppm slightly reduced the fruit yield, but this decrease did not reach the level of significance under both investigated levels of irrigation in both seasons. On the other hand, plants exposed to water deficit and the foliar application of MT at 20 or 40 ppm revealed several positive influences on fruit quality in terms of its content from total soluble solids (TSS), ascorbic acid, and lycopene compared to the untreated plants in both seasons.

## 4. Discussion

In this study, our results indicated that water deficit inhibited plant growth in terms of shoot fresh and dry weights and total chlorophyll compared to plants that were received adequate water supply. It is well documented that water deficit could induce stomatal closure [60], releasing excessive ROS [12]. Which, in turn, leading to the direct suppression of photosynthesis [13] and chlorophyll degradation. Moreover, water deficit can reduce plant growth and negatively affect development by reducing division, differentiation, and cell enlargement [8,61].

Plants resist the dehydration of their leaf tissues by the accumulation of several organic substances and osmolytes. These molecules play a vital role in the osmotic adjustment and uptake of water under drought conditions [62]. Among these substances, the accumulation of proline and soluble sugars in the leaves was investigated in this study; our results indicated that water deficit caused an increase in proline and soluble sugars compared to the well-watered plants in both seasons. These results were in agreement with several previous reports [11,18].

Melatonin (MT) is considered a new plant growth regulator with multiple physiological functions, including seed germination and photosynthesis, and is involved in alleviating the oxidative damages caused by different stresses [25]. In this study, applied-MT generally enhanced the vegetative growth and chlorophyll concentration of tomato plants. Several lines of evidence indicated that exogenous applied-MT could stimulate plant growth under drought stress in several plant species, including soybean [29], tomato [47], and coffee [39]. These effects could be attributed to enhancing the expression of genes that are implicated in cell division, photosynthesis, and metabolism [29]. Additionally, applied-MT increased the concentration of chlorophyll in the leaves, which sometimes has been roughly doubled after just 24 h of application compared to the untreated plants [31]. The protective role of MT in chlorophyll persevering under abiotic stresses could be attributed to its antioxidant capacity and suppressing the up-regulation of some senescence-associated genes [46,63].

On the other hand, in this study, the decrease in proline and soluble sugars in MT-treated plants could indicate that there is no need to accumulate of these substances. The MT already enhances the turgor pressure of tomato leaf and maintain their water balance using different strategies in this respect. These results were previously confirmed in other plant species such as coffee [39] and maize [10].

It is well documented that water deficit leads to excess cellular levels of reactive oxygen species (ROS), and consequently causes oxidative damage to different plant cell components [12,60,64]. In our study, these effects could explain the excessive increase in H_2_O_2_ on one side and increasing the rate of lipid peroxidation as indicated by MDA on the other side. The MDA content is regarded as a reliable predictor of cellular stability against oxidative damage [65]. The reduction in MDA in drought stress plant after melatonin application may due to the fact that melatonin helps to decrease membrane damage caused by an over-accumulation of ROS [66].

Plants have effective and integrated antioxidant systems that maintain ROS level and protect plants from oxidative stress [12,67]. Ascorbate peroxidase (APX), catalase (CAT), and peroxidase (POX) are considered key enzymes in the ascorbate-glutathione cycle. They are mainly responsible for the detoxification of H_2_O_2_ and defense responses against different stresses [14,68,69]. In this study, increasing the activities of these enzymes under water deficit conditions may reflect the integrated role between these enzymes in the detoxification of ROS under unfavorable conditions.

Melatonin was showed to affect the burst of H_2_O_2_, increasing the activities of antioxidant enzymes, and enhancing the ascorbate–glutathione cycle [46]. In our experiments, applied-MT tends to increase the activities of all studied antioxidant enzymes (APX, CAT and, POX). Several reports indicated that the primary function of MT in plants is improving their antioxidant capacity [25,70,71]. In this context, it was demonstrated that under water deficit stress, applied-MT has further boosted the activities of antioxidant enzymes in many plant species, including tomato [47], coffee [39], and maize [10]. Moreover, melatonin application attenuates the antioxidant generation by both increasing the enzyme activity levels and up-regulation of their gene expression [72]. Finally, Huang and his co-worker [10] suggested that that melatonin can preserve tissue redox homeostasis by activating the antioxidant defense mechanism and consequently increase maize drought tolerance.

The fruit yield of tomato plants was decreased in the water-stressed plants compared to the well-watered ones in both seasons. These results could be explained by that water deficit can restrict photosynthesis by affecting stomatal conductance and CO_2_ assimilation in the Calvin cycle, limiting the supply of adenosine triphosphate (ATP) and hindering the regeneration of RuBP [73]. Moreover, in the present study, it was confirmed that water deficit inhibited the vegetative growth of tomato plants in terms of shoot fresh and dry weights, and negatively affected the total chlorophyll (Figure 1). On the other hand, significant increases in the concentration of TSS, ascorbic acid (AsA), and lycopene were observed under water deficit conditions. This may be due to the reduction in the water content in the fruits, leading to an increase in the concentration of the internal components, as is known with the concentration effect. Furthermore, the progressive rise of ABA, which is considered a common sign of drought stress in several plants, could cause an improvement in the quality of tomato fruits by affecting its soluble sugars [74]. On the other hand, increasing the lycopene under water deficit might also be related to the changes that occurred in ABA, because both carotenoids and ABA have the same biosynthetic pathway [75]. This improvement in the quality of tomato fruits in terms of lycopene, organic acids, and total soluble solids under water shortage was previously confirmed in several studies [76,77,78]. In this study, applied-MT was shown to enhance plant growth, chlorophyll, and antioxidant enzymes. Debnath and his co-author [79] found that melatonin triggers the carotenoids biosynthesis in tomato under stress conditions. Moreover, melatonin could have a critical role in intermediating the accumulation of pigment in fruits. All of these effects could explain the positive effect of applied-MT on the yield of fruits and its quality attributes under the circumstances of this study.

## 5. Conclusions

Despite several lines of evidence having confirmed that exogenous applied-MT can alleviate the deleterious effects of drought stress in many plant species, most of these studies were exclusive on seeds, seedlings, and young plants for a short period of their life cycles. The knowledge of using MT as a potential promising agricultural foliar application to improve crop productivity and quality is still limited under adverse open field conditions. Overall, this study demonstrates that MT could contribute to alleviating the impact of drought stress on tomato plants. These improvements were shown in growth, chlorophyll, antioxidant enzymes. Moreover, the exogenous application of melatonin enhanced fruit yield and its quality attributes, including TSS, ascorbic acid, and lycopene. Meanwhile, a noticeable decrease in proline, soluble sugars and ROS was demonstrated in MT- treated plants. In this respect, the treatment of MT at 20 ppm was more effective in mitigating drought stress than 40 ppm. Our findings could highlight the positive effect of melatonin in open field crops in enhancing the quantity and quality of strategic crops. However, we suggest that two more doses in the flowering and fruit setting stage may maximize its positive impact.

## Figures and Tables

**Table 1:** Chemical properties of the experimental soil in 2018 and 2019 seasons.

Season	Macroelements (%)				pH	Microelements (ppm)		
	N	P	K			Fe	B	Zn
**2018**	0.30	0.21	0.40		8.11	2.87	5.41	2.62
**2019**	0.24	0.28	0.52		7.93	3.31	3.90	3.28
	**CaCO_3_%**	**E.C dS/m**	**Soluble Anions (meq/L.)**			**Soluble Cations (meq/L.)**		
			**HCO_3_^−^**	**SO_4_^−2^**	**Cl^−^**	**Ca^++^**	**Mg^++^**	**Na^+^**
**2018**	1.45	1.22	4.23	2.20	6.21	8.55	4.08	4.73
**2019**	1.33	1.10	3.27	3.54	4.70	6.42	2.79	4.30

**Table 2:** Field capacity, wilting point and available soil water of the experimental soil in the two seasons of 2018 and 2019.

Depth of Soil (cm)	2018			2019		
	FC%	Wilting Percentage	Available Water%	FC%	Wilting Percentage	Available Water%
0–20	23.28	13.58	9.70	24.90	13.82	11.08
20–40	22.41	12.64	9.77	23.15	12.50	10.65
Average	22.85	13.11	9.74	24.03	13.16	10.87
